# An alternative novel tool for DNA editing without target sequence limitation: the structure-guided nuclease

**DOI:** 10.1186/s13059-016-1038-5

**Published:** 2016-09-15

**Authors:** Shu Xu, Shasha Cao, Bingjie Zou, Yunyun Yue, Chun Gu, Xin Chen, Pei Wang, Xiaohua Dong, Zheng Xiang, Kai Li, Minsheng Zhu, Qingshun Zhao, Guohua Zhou

**Affiliations:** 1 Department of Pharmacology, Jinling Hospital, School of Medicine, Nanjing University, No. 305 Zhongshan East Road, Nanjing, 210002 People’s Republic of China; 2 MOE Key Laboratory of Model Animal for Disease Study, State Key Laboratory of Pharmaceutical Biotechnology, Model Animal Research Center, Nanjing University, 12 Xuefu Road, Pukou High-tech Development Zone, Nanjing, 210061 People’s Republic of China; 3 College of Pharmaceutical Science, Soochow University, No. 199, Renai Road, Suzhou, 215123 People’s Republic of China

**Keywords:** Structure-guided endonuclease, flap endonuclease-1 (FEN-1), *Fok* I, Guide DNA, DNA editing

## Abstract

Engineered endonucleases are a powerful tool for editing DNA. However, sequence preferences may limit their application. We engineer a structure-guided endonuclease (SGN) composed of flap endonuclease-1 (FEN-1), which recognizes the 3′ flap structure, and the cleavage domain of Fok I (Fn1), which cleaves DNA strands. The SGN recognizes the target DNA on the basis of the 3′ flap structure formed between the target and the guide DNA (gDNA) and cut the target through its Fn1 dimerization. Our results show that the SGN, guided by a pair of gDNAs, cleaves transgenic reporter gene and endogenous genes in zebrafish embryonic genome.

**Electronic supplementary material:**

The online version of this article (doi:10.1186/s13059-016-1038-5) contains supplementary material, which is available to authorized users.

## Background

DNA manipulation is crucial in many molecular biology experiments, both in vitro and in vivo. The exquisite accuracy of type II restriction endonucleases (REases) in target nuclease cleavage makes them an indispensable tool in these experiments. To date, over 3700 type II REases have been characterized, but only 262 different sequences are recognized by these enzymes [1]. These limited sequences are insufficient for meeting various requirements in DNA manipulation. To overcome this limitation, several methods have been developed. The first involves the mutation of amino acid sequences in existing REases, such as the *Not* I variant, to increase the specificity of DNA sequence recognition with the aid of protein structure informatics [2]. The second involves the construction of a novel type of IIS enzyme (also called unorthodox enzymes) by combining the target recognition domain with a DNA cleavage domain, as reported for *Tst* I and *Bmr* I [3]. The third involves the formation of a novel nuclease by fusing various motifs that are specific to different DNA sequences to a cleavage domain of a desired restriction endonuclease. For example, the zinc-finger nuclease (ZFN) [4] consists of multiple zinc-finger motifs tethered in a series (forming a zinc finger array) that recognize target DNA sequences and the *Fok* I cleavage domain that can cut DNA at the target site. ZFNs have been successfully used to knock out genes in a variety of organisms and cells [5–9]. However, it is difficult to target any desired DNA sequence, owing to the preference for G-rich consensus sequences and the high cost of identifying an “active” zinc finger array that can recognize the target DNA sequences [10]. Another example is TALEN, which combines multiple transcription activator-like (TAL) motifs [11] with the *Fok* I cleavage domain. TAL motifs can recognize a single base nucleotide; thus, theoretically, the enzyme can recognize any sequence in a target and has been successfully used to knock out genes in a variety of organisms and cells [12–16]. However, TALEN requires a thymine at the 5ʹ end of the target sequence, which is recognized by two amino-terminal cryptic repeat folds [17]. In the last method, RNA-guided endonucleases (RGENs, the CRISPR–Cas system) have been developed that use RNA instead of peptides to recognize the sequence of interest and to guide the nuclease to the cleavage target [18]. Since then, successful genome editing using RGENs has been expanded to the human genome [19–21]. Compared with ZFN and TALEN, RGEN has the advantage of using a short, synthetic RNA molecule, rather than a protein, for sequence recognition. The RGEN target sites are limited by the requirements of the PAM sequence, which is recognized by Cas9 [22]. Altogether, the specificity of DNA sequences may limit the applications of endonucleases in DNA editing.

Therefore, it would be valuable to develop a new endonuclease that is DNA sequence independent and can cleave any desired sequence. To meet this need, the flap endonuclease-1 (FEN-1), which recognizes a specific DNA structure, shows promise [23]. During DNA replication and repair processes, FEN-1 participates in removing RNA primers or damaged DNA [24, 25]. The newly synthesized DNA and the displaced region compete for base pairing with the template strand, thus resulting in the formation of a double-flap structure [26]. The double-flap structure has a single unpaired 3′ nucleotide (3′ flap). *Afu* FEN-1 catalyzes phosphodiester cleavage after binding to the 3′ flap [27]. Additionally, *Fok* I is a IIS type restriction enzyme that consists of an N-terminal DNA recognition domain and a C-terminal cleavage domain (Fn1). The bipartite nature of *Fok* I has led to the development of artificial enzymes with novel specificities [28], such as ZFN and TALEN.

Therefore, in this study, we engineered a structure-guided endonuclease (SGN) composed of FEN-1 to recognize the 3′ flap structure and the cleavage domain of *Fok* I (Fn1) to cleave the DNA strand. The 3′ flap structure was formed between the target and the artificial guide DNA (gDNA). With structure-guided recognition and capture, any desired target DNA can be cleaved by an SGN, without the need to change endonucleases or peptide units (as in type II REases, ZFN, and TALEN) and the limitations associated with the use of RNA molecules (as in RGEN).

## Results

### Construction and expression of SGN

**Fig. 1:**
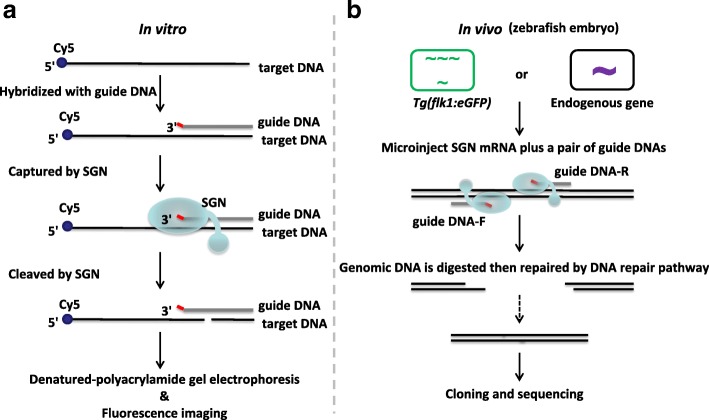
Experimental strategies performed in vitro and in vivo. **a** Experimental strategies performed in vitro. The target DNA was modified with a Cy5 group. The guide DNA was designed complementary to the target with an unpaired nucleotide at the 3′ end to form the 3′ flap structure. The SGN recognizes the 3′ flap and cleaves the target DNA. The cleaved products were analyzed by denatured polyacrylamide gel electrophoresis and fluorescence imaging. **b** Experimental strategies performed in vivo. Here, we used *Tg(flk1 :eGFP)* zebrafish embryos to investigate the SGN activity in vivo. A pair of guide DNAs were designed complementary to the target gene GFP with an unpaired nucleotide at the 3′ end to form the 3′ flap structure. We microinjected the SGN mRNA and the guide DNAs into zebrafish embryos. The expressed SGN recognized the 3′ flap and cleaved the target DNA in vivo. Genomic DNA was digested and repaired by the DNA repair pathway. To examine the DNA editing, we extracted the genomic DNA from the zebrafish embryos and then performed PCR amplification of the GFP target. The amplicons were cloned and sequenced to analyze the mutations caused by SGN. Similar experiments were performed to target the endogenous genes of wild-type zebrafish embryos

We engineered a structure-guided endonuclease (SGN) composed of FEN-1 to recognize the 3′ flap structure and the cleavage domain of *Fok* I (Fn1) to cleave the DNA strand (the coding sequence and amino acid sequence of the SGN are shown in Additional file 1: Figure S1. The plasmid map of pET28a(+)-SGN is shown in Additional file 1: Figure S2). We hypothesized that once the target is recognized by FEN-1, the Fn1 domain of the SGN should be able to cut the DNA strand. Strategies used to validate whether the SGN could cleave the DNA target both in vivo and in vitro are shown in Fig. 1. In vitro (Fig. 1, left), the target DNA was modified with a Cy5 group. The gDNA was designed complementary to the target with an unpaired nucleotide at the 3′ end to form the 3′ flap structure. The SGN recognized the 3′ flap and cleaved the target DNA. The cleaved products were analyzed by denatured-polyacrylamide gel electrophoresis (denatured-PAGE) and fluorescence imaging. Then, we used *Tg(flk1:eGFP)* zebrafish embryo to investigate the SGN activity in vivo (Fig. 1, right). A pair of gDNAs were designed complementary to the target gene GFP with an unpaired nucleotide at the 3′ end to form the 3′ flap structure. The messenger RNA (mRNA) of SGN (with nuclear localization signal) and the guide DNAs were microinjected into zebrafish embryos. The expressed SGN recognized the 3′ flap and cleaved the target DNA in vivo. Genomic DNA was digested and repaired by the DNA repair pathway. To examine the DNA editing, we extracted the genomic DNA from the zebrafish embryos and then performed polymerase chain reaction (PCR) amplification of GFP target. The amplicons were cloned and sequenced to analyze the mutations caused by SGN. Similar experiments were performed to target endogenous genes of wild-type zebrafish embryos.

### SGN cleaved single-stranded DNA in vitro

**Fig. 2:**
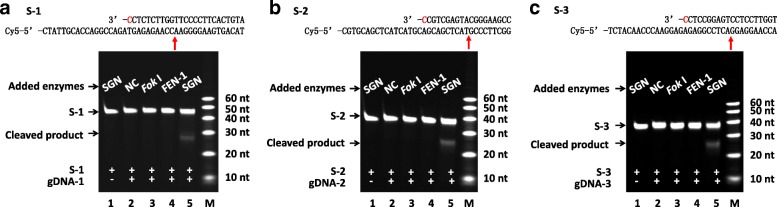
SGN cleaved ssDNA in vitro. Denatured-PAGE showed the DNA products produced by SGN cleavage. **a**–**c** Different target ssDNAs (S-1, S-2, and S-3) were reacted with different gDNAs (gDNA-1, gDNA-2, and gDNA-3). *Lane 1*: S plus SGN; *Lane 2*: S plus gDNA; *Lane 3*: S plus gDNA and *Fok* I; *Lane 4*: S plus gDNA and FEN-1; *Lane 5*: S plus gDNA and SGN. *NC*: no-enzyme control. *Lane M*: DNA standard. The *red arrows* (on the top of each panel) indicate the possible cleavage sites according to the sizes of the cleaved products

To test whether the engineered SGN could cut DNA strands, we incubated SGN with a single-stranded DNA (ssDNA) (S-1) and a gDNA (gDNA-1) (the sequences of all DNA oligos are shown in Additional file 1: Table S1). The S-1 was labeled with a fluorescent Cy5 group on its 5′ end. The gDNA-1 formed a 3′ flap with the S-1. The SGN was added to the mixture to react, and the mixture was then separated by denatured-PAGE and imaged by fluorescence. Theoretically, only the labeled target strand (intact) and the cleaved products containing the 5′ end of S-1 with the fluorescent dye Cy5 should have been visibly detected. As shown in Lane 5 of Fig. 2a, we found that the SGN did cleave the substrate S-1 when guided by gDNA-1, producing bands with smaller sizes denoted by the label “cleaved products.” However, no cleavage occurred in the reactions containing S-1 plus only SGN (Lane 1 in Fig. 2a), S plus only gDNA-1 (Lane 2 in Fig. 2a), S-1 plus *Fok* I and gDNA-1 (Lane 3 in Fig. 2a), or S plus FEN-1 and gDNA-1 (Lane 4 in Fig. 2a). The results demonstrated that the SGN recognized the 3′ flap structure and cleaved the target strand.

### SGN cleavage activity is independent of the target sequence

To clarify whether SGN had a DNA sequence preference, ssDNAs (S-2, S-3) with different sequences (Additional file 1: Table S1) were used as the substrates for SGN. The results revealed that both S-2 and S-3 were cut by SGN (Lane 5 in Fig. 2b and Fig. 2c) when guided by gDNA-2 and gDNA-3, respectively. However, no cleavage occurred in the reactions containing S plus only SGN (Lane 1 in Fig. 2b and Fig. 2c), S plus only gDNA (Lane 2 in Fig. 2b and Fig. 2c), S plus *Fok* I and gDNA (Lane 3 in Fig. 2b and Fig. 2c), or S plus FEN-1 and gDNA (Lane 4 in Fig. 2b and Fig. 2c). The results suggested that the SGN cleavage activity was independent of the target sequence but dependent on the 3′ flap structure.

**Fig. 3:**
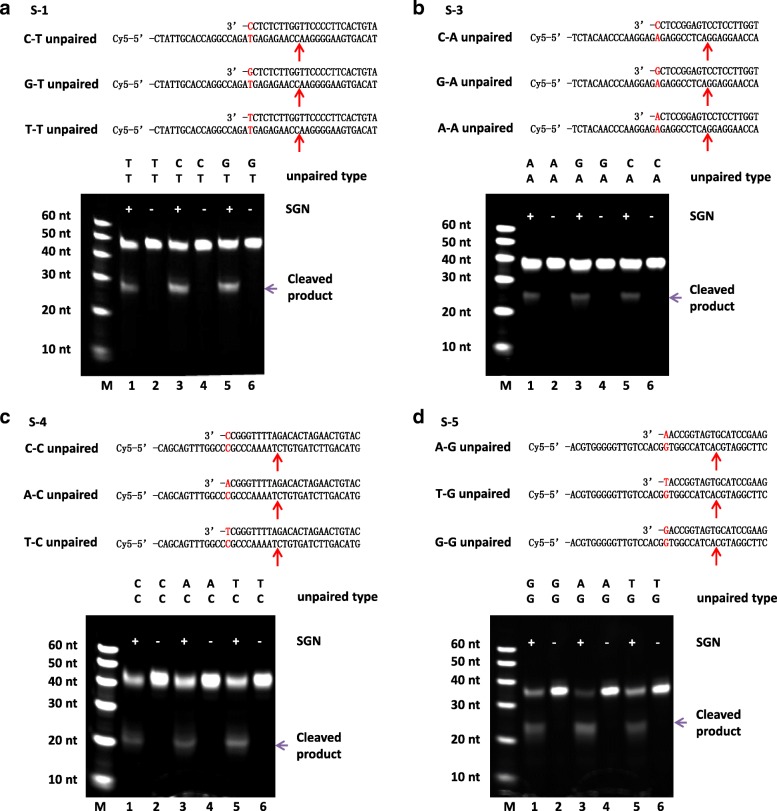
The effect of different unpaired 3′ nucleotides on the DNA cleavage created by SGN. *Schematic* (*top*) shows the unpaired 3′ nucleotide of gDNA on the ssDNA target. The *red arrows* indicate the possible cleavage sites according to the sizes of the cleaved products. The denatured-PAGE results (*below*) show the clearly cleaved products by SGN without obvious difference among the efficiency of the different unpaired types. (**a**) S-1 ssDNA target with gDNA-1, gDNA-1-G, and gDNA-1-T; (**b**) S-3 ssDNA target with gDNA-3, gDNA-3-G, and gDNA-3-A; (**c**) S-4 ssDNA target with gDNA-4, gDNA-4-A, and gDNA-4-T; and (**d**) S-5 ssDNA target with gDNA-5, gDNA-5-T, and gDNA-5-G

**Fig. 4:**
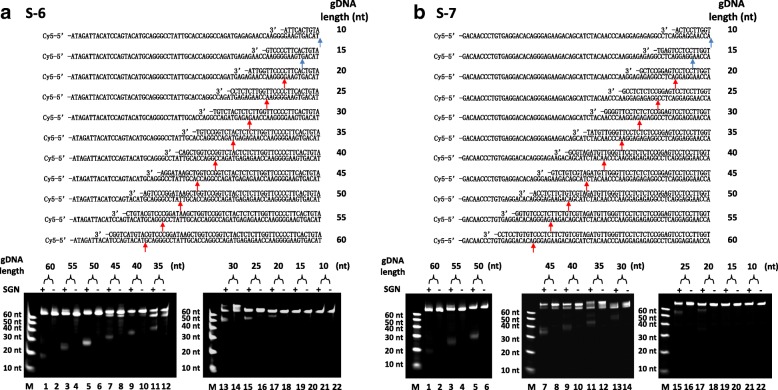
The effect of the length of the gDNA on the DNA cleavage by SGN. *Schematic* (*top*) shows the gDNAs next to the ssDNA targets. The *red arrows* indicate the possible cleavage sites according to the cleaved products sizes. The *blue arrows* indicate the theoretical cleavage sites. The denatured-PAGE results (*bottom*) show the clearly cleaved products by SGN. (**a**) S-6 ssDNA target with gDNA-6-10 nt, gDNA-6-15 nt, gDNA-6-20 nt, gDNA-6-25 nt, gDNA-6-30 nt, gDNA-6-35 nt, gDNA-6-40 nt, gDNA-6-45 nt, gDNA-6-50 nt, gDNA-6-55 nt, and gDNA-6-60 nt; (**b**) S-7 ssDNA with gDNA-7-10 nt, gDNA-7-15 nt, gDNA-7-20 nt, gDNA-7-25 nt, gDNA-7-30 nt, gDNA-7-35 nt, gDNA-7-40 nt, gDNA-7-45 nt, gDNA-7-50 nt, gDNA-7-55 nt, and gDNA-7-60 nt

To address the importance of unpaired 3′ nucleotides in gDNA, we tested all types of unpaired 3′ nucleotides, including C-T, G-T, T-T, C-A, G-A, A-A, C-C, and G-G. As shown in Fig. 3, there was no obvious difference among the efficiency of different unpaired types. As previously reported [24], the archaeal FEN-1 enzymes used all four natural bases with approximately equal efficiency. Our results were consistent with this finding. Then, we tested more sequences to address the importance of gDNA length, including 10, 15, 20, 25, 30, 35, 40, 45, 50, 55, and 60 nucleotides. As shown in Fig. 4, when the length of the gDNAs was more than 20 nt, SGN cleaved the target DNAs. However, when the length of the gDNAs was 10 nt or 15 nt, no cleavage occurred. According to the study on *Fok* I structure [29], the DNA binding to *Fo*k I is ~20 bp. This result indicated that the protein needs enough conformation space on DNA substrate to fold and react. However, as the blue arrows shown in Fig. 4, when the length of the gDNAs was 10 nt or 15 nt, the theoretical cutting sites were too close to the 3′ end of target DNA. Perhaps there is no enough space for conformation requirements in the protein folding process.

### SGN works as a dimer and cleaves a site 9–10 nt away from the unpaired 3′ end

**Fig. 5:**
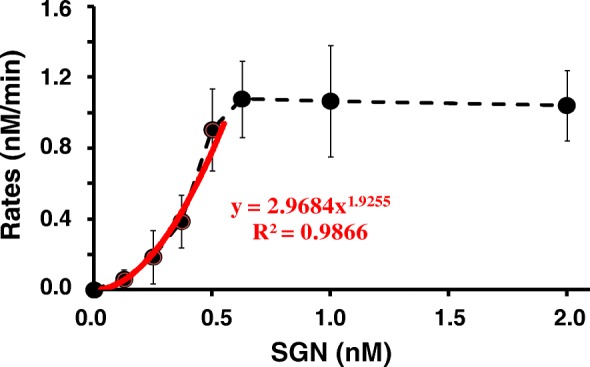
Rates of DNA cleavage with various SGN concentrations. A set of SGN (0, 0.13, 0.25, 0.37, 0.50, 0.62, 1.0, and 2.0 nM) was used to cleave S-8 at a fixed concentration (100 nM). The rates (v) for the cleavage, determined from the generation of the product (P) over time (t), were plotted versus the corresponding SGN concentrations. The rates were fitted into a *red curve* with the equation of *y = 2.9684 x* ^*1.9255*^ before reaching a plateau. The *black dotted curve* linked the average rates of reactions under different concentrations of SGN

It has previously been demonstrated that active *Fok* I works as a dimer [29]. To investigate whether SGN also works as a dimer, we conducted the kinetics experiment of DNA cleavage by SGN. To perform the experiment, we labeled the substrate, S-8, with the fluorescent dye FAM at the 5′ end and the quencher at the 3′ end and incubated it with SGN and gDNA-8. By plotting the rates versus the various concentrations of SGN (Fig. 5), we found that the initial velocity of the reaction was not proportional to the enzyme concentration, thus suggesting that the SGN-catalyzed reaction is not a first-order reaction with respect to the concentration of SGN and implying that SGN works as a dimer. Additionally, SGN is a fusion protein of FEN-1 that is responsible for recognizing the 3′ flap structure and the *Fok* I cleavage domain (Fn1) is responsible for DNA cleavage. As previously reported [29], *Fok* I cuts target DNA through its dimerization, which is mediated through the cleavage domain. In the *Fok* I working model [29], one *Fok* I molecule binds to the recognition site and recruits another *Fok* I molecule, which supplies the second catalytic center through its Fn1 domain. The Fn1 of the first *Fok* I molecule is activated upon binding of specific DNA and swings into an open conformation for dimerization and cleavage. Therefore, we inferred that the dimerization of SGN is also mediated through Fn1.

**Fig. 6:**
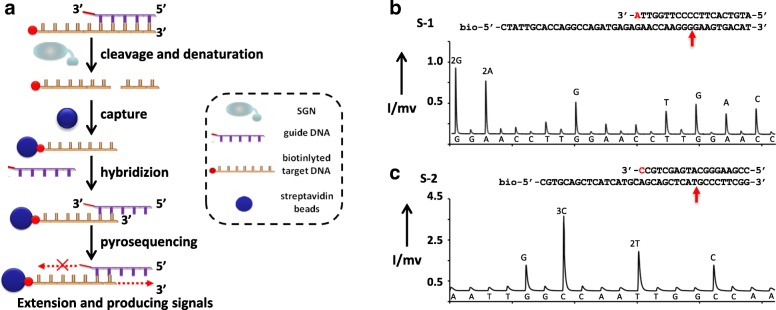
Determination of the cleavage sites. **a** *Schematic* for determining the cleavage sites. The *gray line* represents the probe gDNA (the *black region* represents the one mismatched base at the 3′ end of the gDNA). **b** The *pyrogram* shows the sequencing signal of “GGAAGTGAC.” **c** The *pyrogram* shows the sequencing signal of “GCCCTTC.” The identified cutting sites in (**b**) and (**c**) were marked with a *red arrow* in the inset

To determine the location of the cleavage site created by SGN, we used pyrosequencing [30, 31] to sequence the cleaved strands (Fig. 6a). The targets (S-1 and S-2) were modified with biotin at the 5′ end. After the substrate DNAs were incubated with SGN, streptavidin-coated sepharose beads were used to capture the biotinylated reaction products. The immobilized biotinylated strand was then annealed to its gDNA at 85 °C for 5 min and 25 °C for 10 min, and then used as a sequencing template. Extension did not occur with the unpaired 3′ nucleotides. Therefore, the sequencing signals came only from the cleavage site. As shown in Fig. 6b, the sequence obtained from pyrosequencing signal was GGAAGTGAC. The results showed that the cutting site of S-1 by SGN was at 9 nt away from the 3′ end of gDNA-6-20 nt. In performing similar pyrosequencing for S-2 (Fig. 6c), we found that the sequence from pyrosequencing signal was GCCCTTC. The cleavage sites were 10 nt away from the 3′ end of gDNA-2. Consequently, the results indicated 9–10 nt spacing between the cleavage sites and the 3′ end of the gDNAs.

### SGN partially cleaved double-stranded DNA in vitro

Now that the SGN was able to cut the ssDNA in a manner independent of the sequence but dependent on the 3′ flap structure formed by the gDNA, we wondered whether SGN could digest double-stranded DNA (dsDNA) in vitro. To answer this, three targets were tested. As shown in Additional file 1: Figure S3a–c (Lane 1), bands with smaller sizes denoted by the label “*” were observed in the reactions comprising SGN, a pair of gDNA plus the substrate of dsDNA. However, no cleavage occurred in the reactions containing S plus only SGN (Additional file 1: Figure S3a–c (Lane 2)) or S plus only gDNAs (Additional file 1: Figure S3a–c (Lane 3)). The results suggested that SGN, guided by a pair of gDNAs, could partially cleave dsDNA in vitro. In fact, at the working temperature of SGN (37 °C), the hybridizing efficiency of probe and target dsDNA was low in vitro. We consider this to be the major reason for the faint product bands shown in Additional file 1: Figure S3 (Lane 1). But genomic DNA melts regularly in cells, so the gDNA might hybrid to target strand in vivo more easily than in vitro.

### SGN edited genomic DNA in vivo

**Fig. 7:**
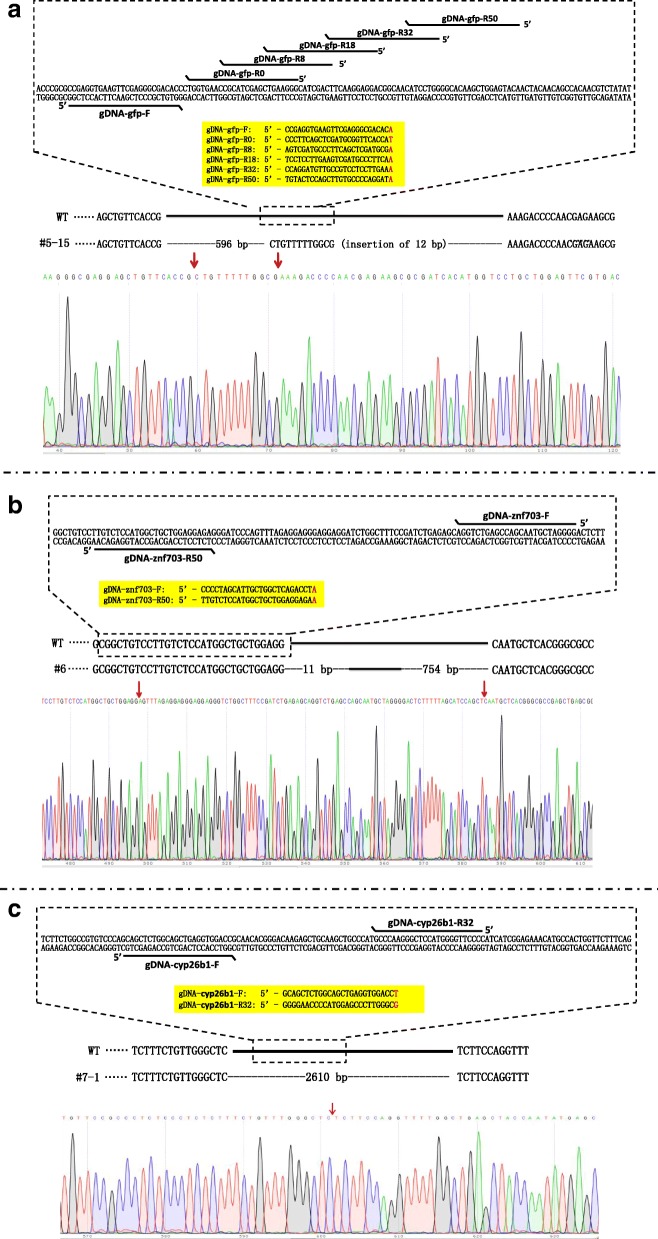
SGN can edit endogenous genes in the zebrafish genome. **a** *Schematic* (*top*) shows the positions of gDNAs on the target *GFP* gene. *Sequencing result* (*bottom*) shows a mutated molecular of #5-15 (Table 1) with a large deletion. **b** *Schematic* (*top*) shows the positions of gDNAs on the target *znf703* gene. *Sequencing result* (*bottom*) from the genome edited products shows that 754 bp were removed with another 11 bp deleted downstream. **c** *Schematic* (*top*) shows the positions of gDNAs on the target *cyp26b1* gene. *Sequencing result* (*bottom*) from the genome edited products shows that 2610 bp were removed. The *red arrows* indicate the positions where large fragment deletion occurs

To examine whether SGN has genome editing activity, we microinjected SGN mRNA and two gDNAs into *Tg*(*flk1:eGFP*) zebrafish embryos at the one-cell stage. The gDNAs were spaced differently, presumably to form the 3′ flap structure with the sense and the antisense strand of dsDNA encoding GFP in the genome of transgenic zebrafish, respectively (Fig. 7a, top). Analysis of the sequences of the genomic target amplified from the embryos microinjected with SGN mRNA plus the gDNA pairs spaced by 0, 8, 18, 32, and 50 bp revealed that 2/48, 0/47, 3/46, 18/44, and 12/47 were mutated molecules, respectively (Table 1 and Additional file 1: Figure S4). The results suggest that SGN has a preference for the genomic sequences with a 32–50 bp spacer. Then, the *znf703* and *cyp26b1* genes were used as targets to validate whether SGN could edit endogenous genes. For the *znf703* gene, analysis of the sequences of the genomic alleles revealed that 1/96 molecule was mutated by SGN guided by gDNA-znf703-F and gDNA-znf703-R50 (Fig. 7b, Table 1, and Additional file 1: Figure S5). The mutated molecule showed that 754 bp was removed and another 11 bp was deleted downstream (Fig. 7b, bottom). For the *cyp26b1* gene (Fig. 7c, Table 1, and Additional file 1: Figure S6), analysis of the sequences of the genomic alleles revealed that 3/29 molecules were mutated by SGN guided by gDNA-cyp26b1-F and gDNA-cyp26b1-R32. The mutated alleles exhibited a large deletion of 2610 bp (Fig. 7c, bottom). The results suggest that SGN can edit endogenous genes of the zebrafish genome, but with low efficiency.

**Table 1:** Mutations of genes in the genome of zebrafish induced by SGN

Target	gDNAs-R	Clone number	Deletion (bp)	Insertion (bp)	Guide DNA spacer (bp)
GFP reporter gene in *Tg(flk1:eGFP)* zebrafish	gDNA-gfp-R0	#1-44	659	3	0
		#1-46	659	3	0
	gDNA-gfp-R8	/	/	/	8
	gDNA-gfp-R18	#3-18	651	1	18
		#3-21	651	1	18
		#3-23	659	3	18
	gDNA-gfp-R32	#4-2	651	1	32
		#4-3	651	1	32
		#4-4	651	1	32
		#4-9	639	17	32
		#4-11	649	20	32
		#4-14	659	3	32
		#4-16	644	0	32
		#4-17	648	1	32
		#4-18	659	3	32
		#4-19	644	0	32
		#4-22	649	20	32
		#4-33	649	20	32
		#4-34	644	0	32
		#4-37	651	1	32
		#4-39	639	17	32
		#4-42	648	1	32
		#4-44	639	17	32
		#4-48	651	1	,
	gDNA-gfp-R50	#5-9	651	0	50
		#5-11	651	0	50
		#5-14	651	0	50
		#5-15	596	12	50
		#5-20	651	0	50
		#5-23	651	0	50
		#5-24	651	0	50
		#5-27	651	0	50
		#5-28	651	0	50
		#5-29	611	3	50
		#5-31	651	0	50
		#5-48	611	3	50
*znf703* endogenous gene	gDNA-znf703-R50	#6	754 + 11	0	50
*cyp26b1* endogenous gene	gDNA-cyp26b1-R32	#7-1	2610	0	32
		#7-2	2610	0	32
		#7-3	2610	0	32

The frequencies of finding a deletion among all the clones: #1 (2/48); #2 (0/47); #3 (3/46); #4 (18/44); #5 (12/47); #6 (1/96); and #7 (3/29)

**Fig. 8:**
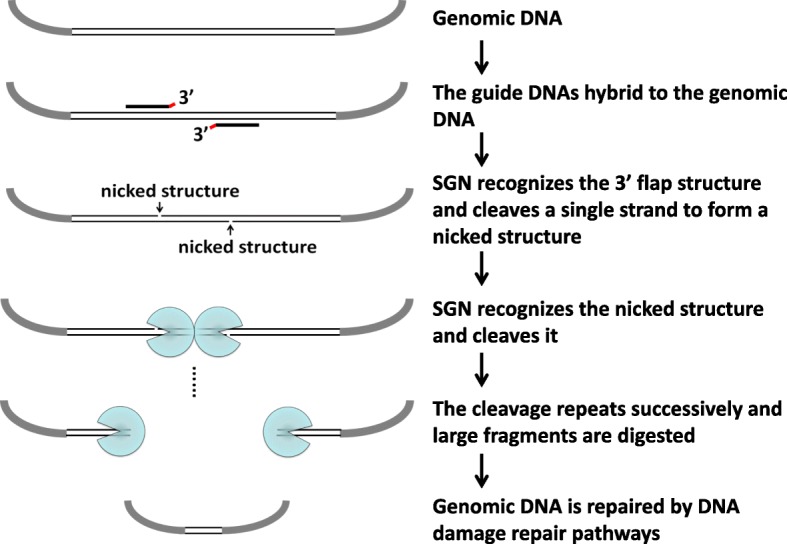
The proposed mechanism underlying large deletions by SGN. One gDNA hybridizes to a single strand of zebrafish genomic DNA to form the 3′ flap-structure. SGN binds to the 3′ flap-structure and cleaves the single strand of zebrafish genomic DNA to create a nicked structure that can be recognized by SGN. One SGN molecule binds to the nicked structure and cleaves the single strand of zebrafish genomic DNA to produce a new nicked structure. This means that once the cleavage starts, it repeats successively; and finally the disrupted genomic DNA is repaired by the DNA repair pathway in vivo

It is known that small indels are the major mutations created by ZFN, TALEN, and RGEN, but SGN creates large deletions. Here, we proposed a hypothesis to explain the mechanism underlying these large deletions. As shown in Fig. 8: (1) one guide DNA hybridizes to a single strand of zebrafish genomic DNA to form the 3′ flap-structure; (2) similarly to the mechanism of cleavage of a single-stranded artificial DNA target, SGN binds to the recognition site and cleaves the single strand of zebrafish genomic DNA; (3) the cleavage of the single strand of zebrafish genomic DNA forms a nicked structure; (4) as reported [32], FEN-1 recognizes the nicked structure. Because SGN recognizes the target by FEN-1, we believe that SGN can recognize a nicked structure; (5) one SGN molecule binds to the nicked structure and cleaves the single strand of genomic DNA. The cleaved product also has a nicked structure. This means that once the cleavage starts, it repeats successively; and (6) the disrupted genomic DNA is repaired by the DNA repair pathway in vivo.

In order to validate the possibility of this mechanism we proposed, nicked DNA was tested. A dsDNA fragment (400 bp) was digested by Nt.BstNBI (a nicking endonuclease, recognition site “GAGTC,” cutting 4 bases behind) to create a dsDNA with a nick in each strand (Additional file 1: Figure S7a). As shown in Additional file 1: Figure S7b (Lane 2), several product bands with smaller sizes were observed from the reaction with both the nicked DNA and SGN, whereas no such results were observed in the nicked DNA without SGN (Additional file 1: Figure S7b (Lane 1)), the dsDNA without SGN (Additional file 1: Figure S7b (Lane 3)), and the dsDNA with SGN (Additional file 1: Figure S7b (Lane 4)). This result provided the preliminary evidence to support the large deletion mechanism we proposed.

## Discussion

Here, we engineered a structure-guided nuclease that recognizes target DNA on the basis of the 3′ flap structure formed between the target and the gDNA and cuts the target through Fn1 dimerization. Currently available endonucleases capture the substrate DNA on the basis of DNA sequence and exhibit sequence preferences. Using structure-guided recognition and capture, we were able to design suitable gDNAs for any desired target DNA and use SGN to cleave targets. It is easy to design and synthesize gDNA and to adjust their concentration, but this is difficult in the RGEN system. Recently, a newly reported study [33] about the NgAgo genome editing system also states the advantage of DNA probes. Moreover, small indels are the major mutations created by ZFN, TALEN, RGEN, and the newly reported NgAgo genome editing system, but a small indel in a mutated gene is still able to encode a truncated protein that usually has some residual function. In other words, the mutated allele with a small indel mutation induced by ZFN, TALEN, RGEN, and NgAgo is not always a null allele or a knockout allele. In contrast, a mutated allele with a large deletion created by SGN is more likely to be a null allele.

Further research is needed because some issues should be resolved before the application of SGN in genomic DNA editing. At present, the efficiency of the SGN system is low, so we did not observe the off-target cases from the *cyp26b1* and *znf703* gene experiments. But according to the SGN working model, we thought the most possible off-target cases would happen due to the mis-hybridization of gDNA with genomic DNA. Fortunately, compared with NgAgo, which only works when its DNA probe is 23 nt, 24 nt, or 25 nt, the length of gDNAs in SGN system could be adjusted to avoid the mis-hybridization. The other potential trigger is that the FEN-1 may target some naturally occurring DNA structures. It perhaps leads to not only off-target effects but also cell toxicity. But in fact we did not observe particular cell death situation in zebrafish experiments. Besides, the inherent challenges of generating a specific structure in dsDNAs other than canonical duplexes cannot be ignored. The efficiency of our method may be severely limited by unlikely interactions between gDNA and the correct site in the genome, thus forming a 3′ flap structure. Other strategies, such as using PNA or LNA probes as gDNA and possible structural modification, may be helpful to improve the cleavage efficiency of SGN.

## Conclusion

In summary, we engineered an SGN composed of FEN-1, which recognizes the 3′ flap structure, and the cleavage domain of *Fok* I (Fn1), which cleaves the DNA strand. The SGN recognizes the target DNA on the basis of the 3′ flap structure formed between the target and the gDNA and cuts the target through its Fn1 dimerization. Our results showed that the SGN can cleave target DNA in vitro. Using zebrafish embryos as an incubation system, we demonstrated that SGN can edit endogenous genes. With structure-guided recognition and capture, any desired target DNA can be cleaved by an endonuclease SGN, without the need to change endonucleases or peptide units (as in type II REases, ZFN, and TALEN) and the limitations associated with the use of RNA molecules (as in RGEN). It may be a useful alternative tool for DNA editing.

## Methods

### Construction of pET28a(+)-SGN

A concatenated sequence encoding the C terminal of *Fok* I (196 amino acid residues), a linker of Gly-Ser repeats, and the *Afu* FEN-1 enzyme were designed. This combined sequence was synthesized and inserted into the prokaryotic expression plasmid of pET28a(+) to form pET28a(+)-SGN by Generay Biotech (Shanghai, China).

### Expression and purification of SGN

The pET28a(+)-SGN was transformed into host bacteria, *Arctic Express*, with the CaCl_2_-heat-shock method. Briefly, cells were cultured at 37 °C, and induced with IPTG (0.1 mM) at 25 °C for 16 h to express SGN. The induced cells were collected, lysed with ultrasound, and centrifuged. SGN was purified from the crude extract by Ni-affinity chromatography. SGN was then concentrated with ultrafiltration and confirmed with sodium dodecyl sulphate (SDS)-polyacrylamide gel electrophoresis (PAGE; 12 %).

### Cleaving ssDNA with SGN in vitro

A 10-μL reaction mixture consisting of gDNA (10 pmol), substrate ssDNA (10 pmol) (Additional file 1: Table S1), MOPS (10 mM), 0.05 % Tween-20, 0.01 % nonidet P-40, MgCl_2_ (7.5 mM), and SGN (1 ng) was prepared. The 5′ end of the target ssDNA strand was labeled with fluorescent group Cy5. Before SGN was added, the mixture was incubated at 95 °C for 5 min and 55 °C for 10 min. SGN was then added and the reaction was incubated at 37 °C for 2 h.

### Denatured-polyacrylamide gel electrophoresis

The products derived from the reactions were analyzed with PAGE under denaturing conditions. The loading buffer contained 90 % formamide, 0.5 % EDTA, 0.1 % xylene cyanol, and 0.1 % bromophenol blue. Before loading, the sample (20 μL) were incubated for 5 min in boiling water and then cooled on ice. The samples were then loaded on a 20 % PAGE gel at room temperature and run in a buffer that contained urea (8.7 M) and Tris-borate (89 mM). The electrophoresis was run at 9.6 V/cm for 2.5 h. After the electrophoresis, the gel was then immersed in 10 % alcohol for 20 min to immobilization. The gel was imaged by the Tanon 5200 multi fluorescence imaging instrument (Shanghai, China).

### Cleaving dsDNA with SGN in vitro

We first incubated the mixture consisting of 5 pmol of S-1/S-3/S-5 and 5 pmol of S-9 (complementary to S-1)/S-10 (complementary to S-3)/S-11 (complementary to S-5) in a process of 95 °C 3 min, 94 °C 1 min to 22 °C 1 min by cooling down with a rate of 1 °C/min, respectively. In this process, the complementary single strands can hybrid to each other as much as possible. Then, 5 pmol of gDNA-1/gDNA-3/gDNA-5, 5 pmol of gDNA-9/gDNA-10/gDNA-11, 10 mM MOPS, 0.05 % Tween-20, 0.01 % nonidet P-40, and 7.5 mM MgCl_2_ were added in the mixture and incubated at 37 °C for 10 min, respectively. Then, 1 ng of SGN was added to be incubated at 37 °C for 2 h.

### Kinetics experiments of DNA cleavage by SGN

The substrate ssDNA for the experiment, namely S-8 (Additional file 1: Table S1), was labeled with FAM at 5′ end and quencher at 3′ end. Briefly, the 10-μL reaction mixture was made of S-8 (1 pmol), gDNA-8 (1 pmol, gDNA of S-8), MOPS (10 mM), 0.05 % Tween-20, 0.01 % nonidet P-40, MgCl_2_ (7.5 mM), and varied concentration of SGN (0, 0.13, 0.25, 0.37, 0.50, 0.62, 1.0, and 2.0 nM, respectively). The kinetics experiment was performed by real time PCR (MJ Research, USA). The reactions were pre-incubated at 95 °C for 5 min and 55 °C for 10 min before adding SGN. The SGN was added to the reaction, mixed well by pipetting up and down, and incubated at 37 °C for 10 min. The increasing signal of FAM could reflect the cleavage of S-8. The difference between the signal of FAM at 10 min and 1 min showed the different amount of S-8 that had been cleaved. The concentration of cleavage product was divided by the time it took to determine the rates of the cleavage reactions. The rates were plotted versus the varied concentrations of SGN in order to analyze the slope of the line.

### Pyrosequencing

Pyrosequencing was performed in a portable bioluminescence analyzer (Hitachi, Ltd., Japan), as previously described [30, 31]. Briefly, streptavidin-coated sepharose beads were used to capture biotinylated reaction products. After sedimentation and washing, purified dsDNAs were denatured in an alkali buffer to yield ssDNAs. The immobilized biotinylated strand was then annealed to a sequencing primer at 85 °C for 5 min and 25 °C for 10 min and then used as a sequencing template. The pyrosequencing mixture contained Tris-HAc (0.1 M, pH 7.7), EDTA (2 mM), Mg(Ac)_2_ (10 mM), 0.1 % BSA, DTT (1 mM), APS (2 μM), PVP (0.4 g L^–1^), D-luciferin (0.4 mM), ATP sulfurylase (2 μM), apyrase-VII (1.6 U mL^–1^), Exo Klenow fragment (18 U mL^–1^), and 5.7 × 10^8^ RLU QuantiLum recombinant luciferase.

### Editing the reporter *eGFP* gene with SGN in *Tg*(*flk1*:*eGFP*) transgenic zebrafish embryos

We microinjected 1 nL of a solution containing 200 pg of SGN mRNA (containing the sequence for encoding nuclear localization signal), plus 50 pg of each gDNA pairs into *Tg*(*flk1*:*eGFP*) transgenic zebrafish embryos at the one-cell stage. The mutated *GFP* molecules were examined with the same method as previously described [34]. Briefly, zebrafish genomic DNA templates were prepared from five 6 hpf embryos randomly selected by incubating the embryos with 10-μL B solution under a program (65 °C for 30 min, 95 °C for 10 min, and 16 °C for 1 min) following the manufacturer’s instructions (Nanjing YSY Biotech, China). One μL of the lysis solution was then used as a template to amplify the *GFP* molecules with the primer pair of GFP F1 and GFP R1 (Additional file 1: Table S1) in a 20-μL PCR mixture. The PCR program was 94 °C for 2 min, 35 cycles of (94 °C for 30 s, 60 °C for 30 s, and 72 °C for 30 s), 72 °C for 10 min. The amplicons were cloned into pGEM-T (Promega, USA). Then, 48 transformants were randomly selected, and positive transformants were identified with the PCR program as described above with the primer pair of GFP F2 and GFP R2 (Additional file 1: Table S1). Next, the target molecules in the positive transformants were sequenced to identify mutations.

### Editing the endogenous genes with SGN in the genomes of zebrafish embryos

To explore whether the SGN system could edit endogenous genes in zebrafish embryos with a certain length spacer like ZFN and TALEN, we microinjected 1 nL of a solution containing 200 pg of SGN mRNA, plus 50 pg of each gDNA pairs into zebrafish embryos at the one-cell stage, respectively. The mutated *znf703* and *cyp26b1* alleles were examined with the similar method as described above. Briefly, 1.0 μL of the lysis solution from five embryos was used as a template to amplify the *znf703* and *cyp26b1* alleles with the primer pairs of znf703 F and znf703 R, cyp26b1 F and cyp26b1 R (Additional file 1: Table S1) in a 20-μL PCR mixture, respectively. The PCR program was 95 °C for 3 min, 30 cycles of (95 °C for 15 s, 60 °C for 15 s, and 72 °C for 1 min 30 s) (for amplifying the alleles of *znf703*) or of (95 °C for 15 s, 52 °C for 15 s, and 72 °C for 3 min) (for amplifying the alleles of *cyp26b1*), 72 °C for 10 min. To increase the specificity of *cyp26b1* amplification, we performed a nested PCR by using a pair of primers of cyp26b1 Fin and cyp26b1 Rin (Additional file 1: Table S1). The amplicons from PCR amplification of *znf703* and nested-PCR amplification of *cyp26b1* were cloned into pGEM-T (Promega, USA). Then, 96 transformants were randomly selected and used as templates to be examined the insert size by PCR amplification with primer pairs of T7 and Sp6 (Additional file 1: Table S1) in a reaction program of 94 °C 2 min, 30 cycles of (94 °C for 30 s, 54 °C for 30 s, and 72 °C for 3 min 10 s), 72 °C for 10 min. The PCR products smaller than predicted wild-type size were diluted ten times and then used as templates to be examined whether they had the insert from mutated alleles of the target genes by PCR amplification with primer pairs of znf703 F and znf703 R, cyp26b1 Fin and cyp26b1 Rin (Additional file 1: Table S1) in the reaction programs as described above. The insert DNA fragments in the positive transformants were further sequenced to identify mutations.

### Cleaving the nicked dsDNA by SGN in vitro

A 50-μL reaction mixture consisting of dsDNA (1 μg), 10 × NEB buffer 3 (5 μL) and Nt.BstNBI (1 μL, NEB) was prepared and incubated at 37 °C for 2 h. The nicked dsDNA fragment was purified by DNA purification kit (TransGen Biotech, China). A 10-μL reaction mixture consisting of purified nicked/nick dsDNA (100 ng), MOPS (10 mM), 0.05 % Tween-20, 0.01 % nonidet P-40, MgCl_2_ (7.5 mM), and SGN (1 ng) was incubated at 37 °C for 2 h. The cleaved products were analyzed by 2 % agarose gel.

## Additional file

Additional file 1: Supplemental Table and Figures. Supplemental Table S1 and Figures S1–S7. (PDF 742 kb)

## References

[1] Jurėnaitė-Urbanavičienė S, Šerkšnaitė J, Kriukienė E, Giedrienė J, Venclovas Č, Lubys A (2007). Generation of DNA cleavage specificities of type II restriction endonucleases by reassortment of target recognition domains. Proc Natl Acad Sci U S A.

[2] Buchholz F (2009). Engineering DNA, processing enzymes for the postgenomic era. Curr Opin Biotech.

[3] Chan S, Bao YE, Laget, Xu S (2007). Catalytic domain of restriction endonuclease Bmrl as a cleavage module for engineering endonucleases with novel substrate specificities. Nucleic Acids Res.

[4] Kim Y-G, Cha J, Chandrasegaran S (1996). Hybrid restriction enzymes: zinc finger fusions to Fok I cleavage domain. Proc Natl Acad Sci U S A.

[5] Bibikova M, Golic M, Golic KG, Carroll D (2002). Targeted chromosomal cleavage and mutagenesis in Drosophila using zinc-finger nucleases. Genetics.

[6] Li H, Haurigot V, Doyon Y, Li T, Wong SY, Bhagwat AS (2011). In vivo genome editing restores haemostasis in a mouse model of haemophilia. Nature.

[7] Straimer J, Lee MC, Lee AH, Zeitler B, Williams AE, Pearl JR (2012). Site-specific genome editing in Plasmodium falciparum using engineered zinc-finger nucleases. Nat Methods.

[8] Urnov FD, Rebar EJ, Holmes MC, Zhang HS, Gregory PD (2010). Genome editing with engineered zinc finger nucleases. Nat Rev Genet.

[9] Kim JS, Lee HJ, Carroll D (2010). Genome editing with modularly assembled zinc-finger nucleases. Nat Methods.

[10] Isalan M (2012). Zinc-finger nucleases: how to play two good hands. Nat Methods.

[11] Li L, Atef A, Piatek A, Ali Z, Piatek M, Aouida M (2013). Characterization and DNA-binding specificities of Ralstonia TAL-like effectors. Mol Plant.

[12] Bloom K, Ely A, Mussolino C, Cathomen T, Arbuthnot P (2013). Inactivation of hepatitis B virus replication in cultured cells and in vivo with engineered transcription activator-like effector nucleases. Mol Ther.

[13] Christian M, Cermak T, Doyle EL, Schmidt C, Zhang F, Hummel A (2010). Targeting DNA double-strand breaks with TAL effector nucleases. Genetics.

[14] Sung YH, Baek IJ, Kim DH, Jeon J, Lee J, Lee K (2013). Knockout mice created by TALEN-mediated gene targeting. Nat Biotechnol.

[15] Tesson L, Usal C, Menoret S, Leung E, Niles BJ, Remy S (2011). Knockout rats generated by embryo microinjection of TALENs. Nat Biotechnol.

[16] Mussolino C, Morbitzer R, Lütge F, Dannemann N, Lahaye T, Cathomen T (2011). A novel TALE nuclease scaffold enables high genome editing activity in combination with low toxicity. Nucleic Acids Res.

[17] Mak AN, Bradley P, Cernadas RA, Bogdanove AJ, Stoddard BL (2012). The crystal structure of TAL effector PthXo1 bound to its DNA target. Science.

[18] Horvath P, Barrangou R (2010). CRISPR/Cas, the immune system of bacteria and archaea. Science.

[19] Cho SW, Kim S, Kim JM, Kim J-S (2013). Targeted genome engineering in human cells with the Cas9 RNA-guided endonuclease. Nat Biotechnol.

[20] Cong L, Ran FA, Cox D, Lin S, Barretto R, Habib N (2013). Multiplex genome engineering using CRISPR/Cas systems. Science.

[21] Mali P, Yang L, Esvelt KM, Aach J, Guell M, DiCarlo JE (2013). RNA-guided human genome engineering via Cas9. Science.

[22] Mojica FJ, Diez-Villasenor C, Garcia-Martinez J, Almendros C (2009). Short motif sequences determine the targets of the prokaryotic CRISPR defence system. Microbiology.

[23] Harrington JJ, Lieber MR (1994). The characterization of a mammalian DNA structure-specific endonuclease. EMBO J.

[24] Kaiser MW, Lyamicheva N, Ma W, Miller C, Neri B, Fors L (1999). A comparison of eubacterial and archaeal structure-specific 5′-exonucleases. J Biol Chem.

[25] Kao HI, Henricksen LA, Liu Y, Bambara RA (2002). Cleavage specificity of Saccharomyces cerevisiae flap endonuclease 1 suggests a double-flap structure as the cellular substrate. J Biol Chem.

[26] Reynaldo LP, Vologodskii AV, Neri BP, Lyamichev VI (2000). The kinetics of oligonucleotide replacements. J Mol Biol.

[27] Chapados BR, Hosfield DJ, Han S, Qiu J, Yelent B, Shen B (2004). Structural basis for FEN-1 substrate specificity and PCNA-mediated activation in DNA replication and repair. Cell.

[28] Li L, Wu LP, Chandrasegaran S (1992). Functional domains in Fok I restriction endonuclease. Proc Natl Acad Sci U S A.

[29] Wah DA, Bitinaite J, Schildkraut I, Aggarwal AK (1998). Structure of FokI has implications for DNA cleavage. Proc Natl Acad Sci U S A.

[30] Zhou G, Kajiyama T, Gotou M, Kishimoto A, Suzuki S, Kambara H (2006). Enzyme system for improving the detection limit in pyrosequencing. Anal Chem.

[31] Wu H, Wu W, Chen Z, Wang W, Zhou G, Kajiyama T (2011). Highly sensitive pyrosequencing based on the capture of free adenosine 5′ phosphosulfate with adenosine triphosphate sulfurylase. Anal Chem.

[32] Hosfield DJ, Frank G, Weng Y, Tainer JA, Shen B (1998). Newly discovered archaebacterial flap endonucleases show a structure-specific mechanism for DNA substrate binding and catalysis resembling human flap endonuclease-1. J Biol Chem.

[33] Gao F, Shen XZ, Jiang F, Wu Y, Han C (2016). DNA-guided genome editing using the Natronobacterium gregoryi Argonaute. Nat Biotechnol.

[34] Dong Z, Ge J, Li K, Xu Z, Liang D, Li J (2011). Heritable targeted inactivation of myostatin gene in yellow catfish (Pelteobagrus fulvidraco) using engineered zinc finger nucleases. PLoS One.

