# Supplementary material for: An alternative novel tool for DNA editing without target sequence limitation: the structure-guided nuclease
Source: Genome Biol. 2016 Sep 15;17:186. doi: 10.1186/s13059-016-1038-5 (PMC5025552; doi:10.1186/s13059-016-1038-5)

Table S1. Sequences of oligodeoxynucleotide templates, guide DNAs and PCR primers

Name of oligodeoxynucleotide	Sequence (5'-3')
gDNA-1	atgtcacttccccttggttctctcc
gDNA-1-G	atgtcacttccccttggttctctcg
gDNA-1-T	atgtcacttccccttggttctctct
S-1	Cy5-ctattgcaccaggccagatgagagaaccaaggggaagtgacat
gDNA-2	ccgaagggcatgagctgcc
S-2	Cy5-cgtgcagctcatcatgcagcagctcatgcccttcgg
gDNA-3	tggttcctcctgaggcctcc
gDNA-3-G	tggttcctcctgaggcctcg
gDNA-3-A	tggttcctcctgaggcctca
S-3	Cy5-tctacaacccaaggagagaggcctcaggaggaacca
gDNA-4	catgtcaagatcacagattttgggcc
gDNA-4-A	catgtcaagatcacagattttgggca
gDNA-4-T	catgtcaagatcacagattttgggct
S-4	Cy5-cagcagtttggcccgcccaaaatctgtgatcttgacatg
gDNA-5	gaagcctacgtgatggccaa
gDNA-5-T	gaagcctacgtgatggccat
gDNA-5-G	gaagcctacgtgatggccag
S-5	Cy5-acgtgggggttgtccacggtggccatcacgtaggcttc
gDNA-6-10nt	atgtcactta
gDNA-6-15nt	atgtcacttcccctg
gDNA-6-20nt	atgtcacttccccttggtta
gDNA-6-25nt (same as gDNA-1)	atgtcacttccccttggttctctcc
gDNA-6-30nt	atgtcacttccccttggttctctcatctgt
gDNA-6-35nt	atgtcacttccccttggttctctcatctggcctgt
gDNA-6-40nt	atgtcacttccccttggttctctcatctggcctggtgcac
gDNA-6-45nt	atgtcacttccccttggttctctcatctggcctggtgcaatagga
gDNA-6-50nt	atgtcacttccccttggttctctcatctggcctggtgcaataggccctga
gDNA-6-55nt	atgtcacttccccttggttctctcatctggcctggtgcaataggccctgcatgtc
gDNA-6-60nt	atgtcacttccccttggttctctcatctggcctggtgcaataggccctgcatgtactggc
S-6	Cy5-atagattacatccagtacatgcagggcctattgcaccaggccagatgagagaaccaaggggaagtgaca t
gDNA-7-10nt	tggttcctca
gDNA-7-15nt	tggttcctcctgagt
gDNA-7-20nt (same as gDNA-3)	tggttcctcctgaggcctcc
gDNA-7-25nt	tggttcctcctgaggcctctctccg
gDNA-7-30nt	tggttcctcctgaggcctctctccttgggg
gDNA-7-35nt	tggttcctcctgaggcctctctccttgggttgtat
gDNA-7-40nt	tggttcctcctgaggcctctctccttgggttgtagatgcg
gDNA-7-45nt	tggttcctcctgaggcctctctccttgggttgtagatgctgtctg
gDNA-7-50nt	tggttcctcctgaggcctctctccttgggttgtagatgctgtcttctcca
gDNA-7-55nt	tggttcctcctgaggcctctctccttgggttgtagatgctgtcttctccctgtgg

Name of oligodeoxynucleotide	Sequence (5’-3’)
gDNA-7-60nt	tggttcctcctgaggcctctctccttgggttgtagatgctgtcttctccctgtgtcctcc
S-7	Cy5-gacaaccctgtgaggacacagggagaagacagcatctacaacccaaggagagaggcctcaggaggaacca
gDNA-8	ctcctgattatgac
S-8	FAM-tcagccactggcttctgtcataatcaggag-ECLIPSE
gDNA-9	ctattgcaccaggccagc
S-9	atgtcacttccccttggttctctcatctggcctggtgcaatag
gDNA-10	tctacaacccaaggat
S-10	tggttcctcctgaggcctctctccttgggttgtaga
gDNA-11	acgtgggggttgtccact
S-11	gaagcctacgtgatggccaccgtggacaacccccacgt
GFP F1	gtgagcaagggcgaggagctg
GFP R1	cttgtacagctcgtccatgccg
GFP F2	aagggcgaggagctgttcacc
GFP R2	agctcgtccatgccgagagtg
gDNA-gfp-F	ccgaggtgaagttcgagggcgacaca
gDNA- gfp -R0	cccttcagctcgatgcggttcaccat
gDNA- gfp -R8	agtcgatgcccttcagctcgatgcga
gDNA- gfp - R18	tcctccttgaagtcgatgcccttcaa
gDNA- gfp - R32	ccaggatgttgccgtcctccttgaaa
gDNA- gfp - R50	tgtactccagcttgtgccccaggata
gDNA-znf703-F	cccctagcattgctggctcagaccta
gDNA-znf703-R50	ttgtctccatggctgctggaggagaa
znf703 F	tttgcagtcagtccacgg
znf703 R	gactggagttggcgatttgt
gDNA-cyp26b1-F	gcagctctggcagctgaggtggacct
gDNA-cyp26b1-R32	ggggaaccccatggagcccttgggcg
cyp26b1 F	atcggcacaaaaggtaaagc
cyp26b1 R	atgatcttatgtggcacagtc
cyp26b1 Fin	attcatagaaacgcgcttcac
cyp26b1 Rin	agctcatattggtagctcagc
T7	taatacgactcactataggg
Sp6	atttaggtgacactatag

Figure S1. The coding sequence and amino acid sequence of SGN.

```
                    His-Tag                                           FEN-1
1    ATGggcagcagccatcatcatcatcatcacagcagcggcctggtgccgcgcggcagccatatgggtgcggatattggtgac
1    M G S S H H H H H H S S G L V P R G S H M G A D I G D
82   ctctttgagagggaagaggtcgagcttgagtacttctcaggaaagaaaattgccgttgatgctttcaacacgctataccag
28   L F E R E E V E L E Y F S G K K I A V D A F N T L Y Q
163  ttcatctcgataataaggcagcctgacggtacgccgttaaaggactcacagggcagaatcacctctcacctttccggaatc
55   F I S I I R Q P D G T P L K D S Q G R I T S H L S G I
244  ctatacagagtctccaacatggtcgaggtgggaatcaggccggtgtttgtattcgacggagagccaccggagttcaagaag
82   L Y R V S N M V E V G I R P V F V F D G E P P E F K K
325  gctgaaattgaggagaggaaaaagagaagggctgaggcagaggagatgtggattgcggctttgcaggcaggagataaggac
109  A E I E E R K K R R A E A E E M W I A A L Q A G D K D
406  gcgaaaaagtatgctcaggctgcagggagggttgacgagtacattgttgactccgcaaagacgctttaagttacatgggg
136  A K K Y A Q A A G R V D E Y I V D S A K T L L S Y M G
487  attccctttgtcgatgccccgtctgaaggagaggcgcaggctgcttacatggcagcaaaggcgatgtggagtacacagga
163  I P F V D A P S E G E A Q A A Y M A A K G D V E Y T G
568  agccaggattacgattctctgctcttcggaagcccgagactcgccagaaatctcgcaataacgggaaaaaggaagcttccc
190  S Q D Y D S L L F G S P R L A R N L A I T G K R K L P
649  ggcaaaaatgtctatgtggatgtaaagccggagataataattctggaaagcaacctcaaaaggctgggtttgacgagggag
217  G K N V Y V D V K P E I I I L E S N L K R L G L T R E
730  cagctcatcgacatagcgattctggtcgggacggactacaatgagggtgtgaagggtgtcggcgtcaagaaggctttgaac
244  Q L I D I A I L V G T D Y N E G V K G V G V K K A L N
811  tacatcaagacctacggagatattttcagggcactcaaggctctgaaagtaaatattgaccacgtagaggagataaggaat
271  Y I K T Y G D I F R A L K A L K V N I D H V E E I R N
892  ttcttcctgaatcctcctgtgactgacgactacagaatagagttcagggagcctgactttgagaaggccatcgagttcctg
298  F F L N P P V T D D Y R I E F R E P D F E K A I E F L
973  tgcgaggagcacgacttcagcagggagagggtcgagaaggccttggagaagctcaaagctctgaagtcaacccaggccacg
325  C E E H D F S R E R V E K A L E K L K A L K S T Q A T

                                              Linker                   Fn1
1054 cttgaggtggttcgaattcggcggcggcggcagtggaggaggaggaagcggtggtggtggtagtcaactagtcaaaagt
352  L E R W F E F G G G G S G G G G S G G G G S Q L V K S
1135 gaactggaggagaagaaatctgaacttcgtcataaattgaaatatgtgcctcatgaatatattgaattaattgaaattgcc
379  E L E E K K S E L R H K L K Y V P H E Y I E L I E I A
1216 agaaattccactcaggatagaatccttgaaatgaaggtaatggaatttttttatgaaagtttatggatatagaggtaaacat
406  R N S T Q D R I L E M K V M E F F M K V Y G Y R G K H
1297 ttgggtggatcaaggaaaccggacggagcaatttatactgtcggatctcctattgattacggtgtgatcgtggatactaaa
433  L G G S R K P D G A I Y T V G S P I D Y G V I V D T K
1378 gcctatagcggaggttataatctgccaattggccaagcagatgaaatgcaacgatatgtcgaagaaaatcaaacacgaaac
460  A Y S G G Y N L P I G Q A D E M Q R Y V E E N Q T R N
1459 aaacatatcaaccctaatgaatggtggaaagtctatccatcttctgtaacggaatttaagttttttatttgtgagtggtcac
487  K H I N P N E W W K V Y P S S V T E F K F L F V S G H
1540 tttaaaggaaactacaaagctcagcttacacgattaaatcatatcactaattgtaatggagctgttcttagtgtagaagag
514  F K G N Y K A Q L T R L N H I T N C N G A V L S V E E
1621 cttttaattggtggagaaatgattaaagccggcacattaaccttagaggaagtgagacggaaatttaataacggcgagata
541  L L I G G E M I K A G T L T L E E V R R K F N N G E I
1702 aactttaagctttga
568  N F K L
```

Figure S2. The plasmid map of plasmid pET28a(+)-SGN. In the construct, the SGN gene was placed into the downstream of the T7 promoter.

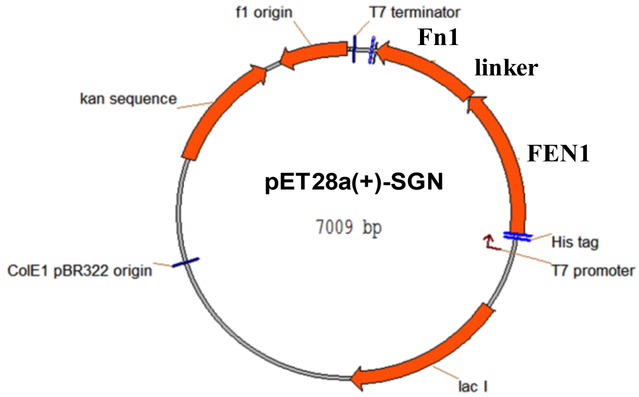

Figure S3. SGN cleaved double-stranded DNA *in vitro*. Denatured PAGE gel shows the cleaved products. a-c: Different target double-strand DNAs (S-1/S-9, S-3/S-10 and S-5/S-11) were reacted with different guide DNAs. Lane 1: S plus SGN and gDNA; Lane 2: S plus SGN; Lane 3: S plus gDNA; Lane M: DNA standard. *： denoting the cleaved product.

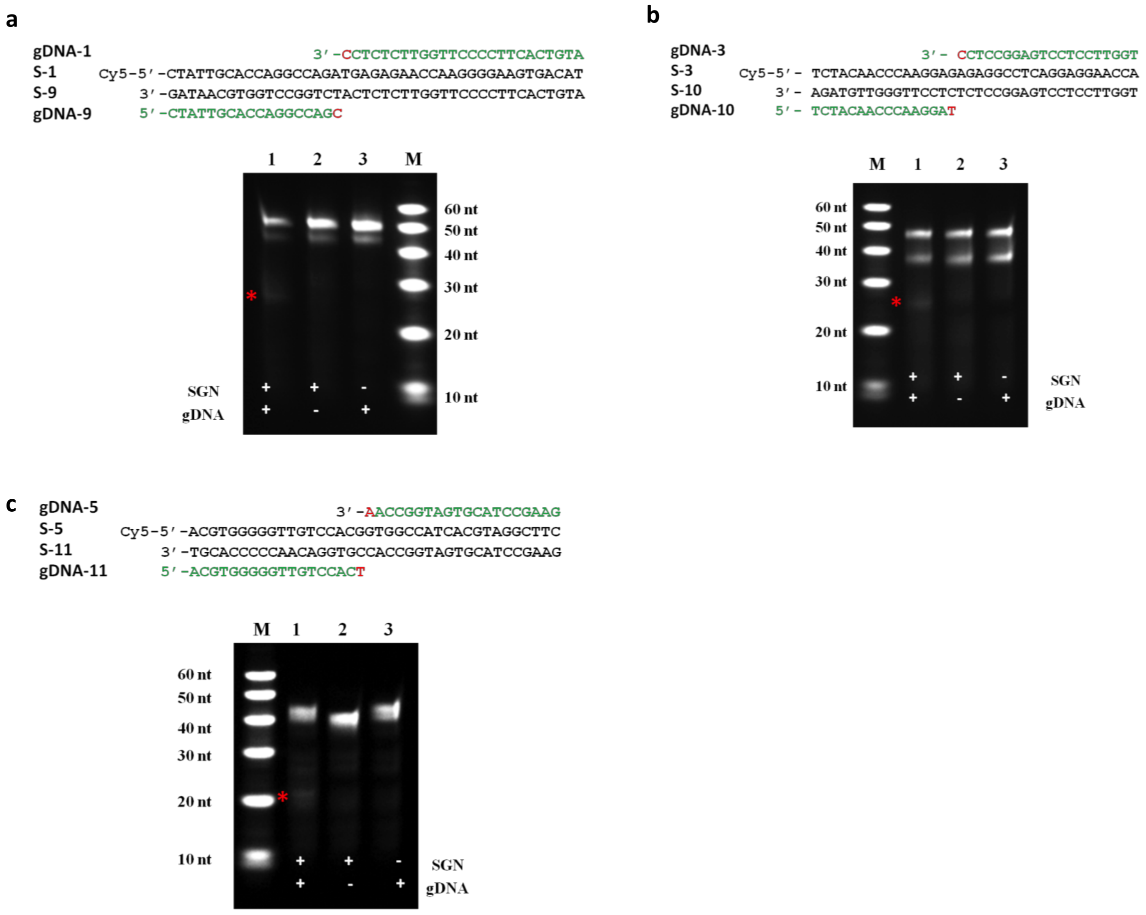

Figure S4. The mutated sequences of *gfp* reporter gene in Tg(*flk*:*eGFP*) transgenic zebrafish created by DNA-guided SGN.

The coding sequence of *eGFP* (720 bp):

ATGGTGAGCAAGGGCGAGGAGCTGTTCACCGGGGTGGTGCCCATCCTGGTCGAGCTGGACGGCGACGTAAACGGCCACA
AGTTCAGCGTGTCCGGCGAGGGCGAGGGCGATGCCACCTACGGCAAGCTGACCCTGAAGTTCATCTGCACCACCGGCAA
GCTGCCCGTGCCCTGGCCCACCCTCGTGACCACCCTGACCTACGGCGTGCAGTGCTTCAGCCGCTACCCCGACCACATG
AAGCAGCACGACTTCTTCAAGTCCGCCATGCCCGAAGGCTACGTCCAGGAGCGCACCATCTTCTTCAAGGACGACGGCA
ACTACAAGACCCGCGCCGAGGTGAAGTTCGAGGGCGACACCCTGGTGAACCGCATCGAGCTGAAGGGCATCGACTTCAA
GGAGGACGGCAACATCCTGGGGCACAAGCTGGAGTACAACTACAACAGCCACAACGTCTATATCATGGCCGACAAGCAG
AAGAACGGCATCAAGGTGAACTTCAAGATCCGCCACAACATCGAGGACGGCAGCGTGCAGCTCGCCGACCACTACCAGC
AGAACACCCCCATCGGCGACGGCCCCGTGCTGCTGCCCGACAACCACTACCTGAGCACCCAGTCCGCCCTGAGCAAAGA
CCCCAACGAGAAGCGCGATCACATGGTCCTGCTGGAGTTCGTGACCGCCGCCGGGATCACTCTCGGCATGGACGAGCTG
TACAAGTAA

The sequence of mutated molecules derived from the *eGFP* reporter of Tg(*flk*:*eGFP*) transgenic zebrafish:

1-44

GTGAGCAAGGGCGAGGAGCTGTTCACC-----659bp---CTT (insertion of 3bp)-----CACTCTCGGCATGGACGAGCTGTACAAG

1-46

GTGAGCAAGGGCGAGGAGCTGTTCACC-----659bp---CTT (insertion of 3bp)-----CACTCTCGGCATGGACGAGCTGTACAAG

3-18

GTGAGCAAGGGCGAGGAGCTGTTCACCGGGGTG--------651bp---A (insertion of 1bp)-----ATCACTCTCGGCATGGACGAGCTGTACAAG

3-21

GTGAGCAAGGGCGAGGAGCTGTTCACCGGGGTG--------651bp---A (insertion of 1bp)-----ATCACTCTCGGCATGGACGAGCTGTACAAG

3-23

GTGAGCAAGGGCGAGGAGCTGTTCACC-----659bp---CTT (insertion of 3bp)-----CACTCTCGGCATGGACGAGCTGTACAAG

4-2

GTGAGCAAGGGCGAGGAGCTGTTCACCGGGGTG--------651bp---A (insertion of 1bp)-----ATCACTCTCGGCATGGACGAGCTGTACAAG

4-3

GTGAGCAAGGGCGAGGAGCTGTTCACCGGGGTG--------651bp---A (insertion of 1bp)-----ATCACTCTCGGCATGGACGAGCTGTACAAG

4-4

GTGAGCAAGGGCGAGGAGCTGTTCACCGGGGTG--------651bp---A (insertion of 1bp)-----ATCACTCTCGGCATGGACGAGCTGTACAAG

4-9

GTGAGCAAGGGCGAGGAGCTGTTCACCGGGGTGGTGCCCATCC----639bp------ATCCTGTTTGTGCCCCA (insertion of 17bp)-------GGATCACTCTCGGCATGGACGAGCTGTACAAG

4-11

GTGAGCAAGGGCGAGGAGCTGTTCACCGGGGTGGTG------649bp------GCCTAACTACGGCTGCTCGT (insertion of 20bp)-------TCACTCTCGGCATGGACGAGCTGTACAAG

4-14

GTGAGCAAGGGCGAGGAGCTGTTCACC-----659bp---CTT (insertion of 3bp)-----CACTCTCGGCATGGACGAGCTGTACAAG

4-16

GTGAGCAAGGGCGAGGAGCTGTTCACCGGGGTGGTGCCCATC-----644bp------CACTCTCGGCATGGACGAGCTGTACAAG

4-17

GTGAGCAAGGGCGAGGAGCTGTTCACCGGGGTGGTG------648bp-----C (insertion of 1bp)-----ATCACTCTCGGCATGGACGAGCTGTACAAG

4-18

GTGAGCAAGGGCGAGGAGCTGTTCACC-----659bp---CTT (insertion of 3bp)-----CACTCTCGGCATGGACGAGCTGTACAAG

4-19

GTGAGCAAGGGCGAGGAGCTGTTCACCGGGGTGGTGCCCATC-----644bp------CACTCTCGGCATGGACGAGCT GTACAAG

4-22

GTGAGCAAGGGCGAGGAGCTGTTCACCGGGGTGGTG------649bp------GCCTAACTACGGCTGCTCGT(insertion of 20bp)-------TCACTCTCGGCATGGACGAGCTGTACAAG

4-33

GTGAGCAAGGGCGAGGAGCTGTTCACCGGGGTGGTG------649bp------GCCTAACTACGGCTGCTCGT(insertion of 20bp) -------TCACTCTCGGCATGGACGAGCTGTACAAG

4-34

GTGAGCAAGGGCGAGGAGCTGTTCACCGGGGTGGTGCCCATC-----644bp------CACTCTCGGCATGGACGAGCT GTACAAG

4-37

GTGAGCAAGGGCGAGGAGCTGTTCACCGGGGTG--------651bp---A (insertion of 1bp)----- ATCACTCTCGGCATGGACGAGCTGTACAAG

4-39

GTGAGCAAGGGCGAGGAGCTGTTCACCGGGGTGGTGCCCATCC----639bp------ATCCTGTTTGTGCCCCA(insertion of 17bp)-------GGATCACTCTCGGCATGGACGAGCTGTACAAG

4-42

GTGAGCAAGGGCGAGGAGCTGTTCACCGGGGTGGTG------648bp-----C (insertion of 1bp)----- ATCACTCTCGGCATGGACGAGCTGTACAAG

4-44

GTGAGCAAGGGCGAGGAGCTGTTCACCGGGGTGGTGCCCATCC----639bp------ATCCTGTTTGTGCCCCA(insertion of 17bp)-------GGATCACTCTCGGCATGGACGAGCTGTACAAG

4-48

GTGAGCAAGGGCGAGGAGCTGTTCACCGGGGTG--------651bp---A (insertion of 1bp)----- ATCACTCTCGGCATGGACGAGCTGTACAAG

5-9

GTGAGCAAGGGCGAGGAGCTGTTCACCGG----651bp------CGGGATCACTCTCGGCATGGACGAGCTGTACAAG

5-11

GTGAGCAAGGGCGAGGAGCTGTTCACCGG----651bp------CGGGATCACTCTCGGCATGGACGAGCTGTACAAG

5-14

GTGAGCAAGGGCGAGGAGCTGTTCACCGG----651bp------CGGGATCACTCTCGGCATGGACGAGCTGTACAAG

5-15

GTGAGCAAGGGCGAGGAGCTGTTCACCG-------596bp-----CTGTTTTTGGCG (insertion of 12bp)-----AAAGACCCCAACGAGAAGCGCGATCACATGGTCCTGCTGGAGTTCGTGACCGCCGCCGGGATCACTCTCGGCATGGACGAGCTGTACAAG

5-20

GTGAGCAAGGGCGAGGAGCTGTTCACCGGCGGG----651bp------ ATCACTCTCGGCATGGACGAGCTGTACAAG

5-23

GTGAGCAAGGGCGAGGAGCTGTTCACCGGCGGG----651bp------ ATCACTCTCGGCATGGACGAGCT

5-24

GTGAGCAAGGGCGAGGAGCTGTTCACCGGCGGG----651bp------ ATCACTCTCGGCATGGACGAGCTGTACAAG

5-27

GTGAGCAAGGGCGAGGAGCTGTTCACCGGCGGG----651bp------ ATCACTCTCGGCATGGACGAGCTGTACAAG

5-28

GTGAGCAAGGGCGAGGAGCTGTTCACCGGCGGG----651bp------ ATCACTCTCGGCATGGACGAGCTGTACAAG

5-29

GTGAGCAAGGGCGAGGAGCTGTTCACCGGGGCGGTGCCCATC------611bp--------ACC(insertion of 3bp)------GGTCCTGCTGGAGTTCGTGACCGCCGCCGGGATCACTCTCGGCATGGACGAGCTGTACAAG

5-31

GTGAGCAAGGGCGAGGAGCTGTTCACCGGCGGG----651bp------ ATCACTCTCGGCATGGACGAGCTGTACAAG

5-48

GTGAGCAAGGGCGAGGAGCTGTTCACCGGGGCGGTGCCCATC------611bp--------ACC(insertion of 3bp)------GGTCCTGCTGGAGTTCGTGACCGCCGCCGGGATCACTCTCGGCATGGACGAGCTGTACAAG

Figure S5. The mutated sequences of *znf703* gene in zebrafish created by DNA-guided SGN.

Partial sequecne of wild type *znf703*

TTTGCAGTCAGTCCACGGAACCAGCAGACCTTTAAAAGTCATTACATTACCAACGATAGTTCGCCCACTTGTTTAGGAC

TCCAGGTCGACTCGATCGCCTCACTGCCGTACCGTGATCCCGCGCGTCAGGAGAAAAGGCTGCCTATACGGATTCTCAA

AATGTTGACCGCTCACACAAGTCACATACTTCACCCAGAGTATCTCCAGCCGCTCAGCTCGGCGCCCGTGAGCATTGAG

GTACGACTTCTAAACCATTTGTTTCTTACTTCTGCGAAGTAAAAATATATACATTACATTTGTATGTGTTAGAGTTGCG

TGTTTGTCCTAGTTTCTATAAAGCGAAAGGGTGTTCTCTTTATTTTGTGCCCGAGTCGTGTAGTACACGCAATATTTTG

CGTCAACGTTTTTTAGCTCTAAGCTCCATGTTGCGCGGCTCGTTTTAATATGTAATTCGGATTTAGCCGAAAGATCCTG

AAAGCGATTCAGCGGCAGTTTGAAGAAATAGTCCGGATTTAGATGAGGCCGTGCTCTGGCAACATTTTAAGGTTTCACA

TTTCCTTAATCGCTACTGACGTGCGCCTTTACTGCTCTTAGGTGGTTAAAGCTTCCAGCATCTACGTGCTTTTTTGGCA

CACAAATGGAACTTTAAAAAAGTAAATTGTGCAATCTTTTTAATAACCTGTATAAAACTCTGCATGCTCGGCACACTTT

GAGTGCTGCAAAAACTACAACAATATGTCAAGTGATTTAATTCACCCTTGACATGCAAATTAACATCATTTAAAATAAA

ATTTAATATCCATGCGAGTTTTTTATGATGATGATGTCATTTATAAATAATCCAAGCGTAAATAGAATTATTGCATCTT

CCTTGAAATATGACATATCACTGAGGGCATGCCAAAAAAATTGGTCTATAAATGAAGTGTCATTTTGTTGGTGGTAAGAA

AACCAAAATCATTCATTTAAAATTTCTTCTTTTTTTCCCATAGCTGGATGCTAAAAAGAGTCCCCTAGCATTGCTGGCT

CAGACCTGCTCTCAGATCGGAAAGCCAGATCCTCCTCCCTCCTCTAAACTGGGATCCCTCTCCTCCAGCAGCCATGGAG

ACAAGGACAGCCGCTCCAGCAGCTCTAGCCTGAAGTCTGGGGAGCATCAGAACCTAGATGACAAGTCCAGCTTTAAGCC

TTACTCTAAAACTGGATCAGAATGCCGAAAGGAGGGTGCTGGGATCAACAGCTCAGCAGATAAAGCGGGGTTCAGAGTG

CCAAATGGCAGTTCTTCATCTGTGACGTGCACGTCTTTACCTCCGCATGCCCCATCTCCACGGGCCAGCTCCCCTCAGC

AAACATCTGGACAGTCACACACACACCGGCAGTCTCAGTCTCCTCTTTCACAGAAAACAGCACATCTACAAACCACCCA

CATGGACTCCAAAGCTGCAGGTTCAGACCCAGGGAATGACAGCAGCAGCAGTGGTAGTGATCGCAATGGCAAAAAAGAT

TCGGACCACAATAAATCAAGCCTGGACGTTGTACAAATCGCCAACTCCAGTC

Partial sequecne of mutated znf703 created by SGN

#6

TTTGCAGTCAGTCCACGGAACCAGCAGACCTTTAAAAGTCATTACATTACCAACGATAGTTCGCCCACTTGTTTAGGAC

TCCAGGTCGACTCGATCGCCTCACTGCCGTACCGTGATCCCGCGCGTCAGGAGAAAAGGCTGCCTATACGGATTCTCAA

AATGTTGACCGCTCACACAAGTCACATACTTCACCCAGAGTATCTCCAGCCGCTCAGCTCGGCGCCCGTGAGCATTG--

---754bp----AGCTGGATGCTAAAAAGAGTCCCCTAGCATTGCTGGCTCAGACCTGCTCTCAGATCGGAAAGCCAGA

TCCTCCTCCCTCCTCTAAACT-----11bp-----CCTCCAGCAGCCATGGAGACAAGGACAGCCGCTCCAGCAGCTCT

AGCCTGAAGTCTGGGGAGCATCAGAACCTAGATGACAAGTCCAGCTTTAAGCCTTACTCTAAAACTGGATCAGAATGCC

GAAAGGAGGGTGCTGGGATCAACAGCTCAGCAGATAAAGCGGGGTTCAGAGTGCCAAATGGCAGTTCTTCATCTGTGAC

GTGCACGTCTTTACCTCCGCATGCCCCATCTCCACGGGCCAGCTCCCCTCAGCAAACATCTGGACAGTCACACACACAC

CGGCAGTCTCAGTCTCCTCTTTCACAGAAAACAGCACATCTACAAACCACCCACATGGACTCCAAAGCTGCAGGTTCAG

ACCCAGGGAATGACAGCAGCAGCAGTGGTAGTGATCGCAATGGCAAAAAAGATTCGGACCACAATAAATCAAGCCTGGA

CGTTGTACAAATCGCCAACTCCAGTC

Figure S6. The mutated sequences of *cyp26b1* gene in zebrafish created by DNA-guided SGN

Partial sequecne of wild type ***cyp26b1***

ATTCATAGAAACGCGCTTCACAGATCGAAATATTTGGCCATATCAATAATTTTGGCAAGGACTATCTGATATAATTAAG

AAAACACTGCCAGTTTATGAGGGATTTATTTACATTATTTTATAGAAATCGACTTATAACATCAACCCCCGAAAGTTGT

AAATATAAACGCAAAACAAATATGAAACGCGTAAGTCAATGAATGAATCGCTTACTCATGCAGTGTTCAGAGGACTGCT

GCTGCTGCTGCTGCTGCTGCTGCTGTGTGCAACAGATGCGCATCTGAACAATCAAAACATTTTGAAAAAGTCATGAACA

TATCGATTTAGCATAAGAGTCCGTAGCTTATATGATCCGGTAAAATGTAGAGTATTATTTCACATTTTTGTTGTGATGG

TTTTAAACCATCATTTGGGTAATCAGTGTACCTCAGTTTGAGCGCATTTTAAGTCCACAACATGAATTAACCCTGTGCG

TATTCCGCGAGTGGCAGGTGACAAGTTCCCGACCGTAAATGAGATTTGAAATATTGAAAGTGTTCCGCCCTCTCCCTCT

CTTTCTGTTTGGGCTCTCTTCCAGGTCCCGCACGCTCCAGCGCCCGTCCCGTTCACTTTTCACCCCGCCTCAGTTCGGT

GGGGTGGGGTAAGGGGGGAGCCGCTTGCACCCTGGCCGAATGCCGCCTCACTGCCTCTGCTATATAAACACACATTGGA

GGCTCGCTGTGCGACTCGAACTCACATTAAGAAACTGTATGTGAAGAGAGAGAGAGAAATAGAGAGAGAGAGAGAGAAA

GAGAGAGAGAGAGAGCTGGAGCGCAAGAGAGAGGTAGAGAGTGAAACCGCATCAGAAAACGGGCTAGCGAGACGCACTA

CAGTCATCTGAAGATTTTTACTGCTCATAGGCATCAAACTTCGGAGGATTATTTCTTTCATTTATTTCTTCGTCTTCCA

TATGACCATTACTTGATTTCAGTGTATTTAAAGAGTTTATATCGAGCTTTAAGATTATATATAGGCCTATTTAAAAATT

ATAACTAAAACAATTTATTTTATATTTTCAAAAAGTAACTTAAAACTTTTTCTTGATTACTTATATTTGCCTTTTTTAC

TTTTGAAATTTAAAACAACGAATCAATCTCAAGTTGGACAAGTTCACTCCAAATTTACACTTCAAAAATTTGAATTTAA

TTGATGAATTCAAGAAATATTGGTCACCGATTTGGATGATTTAAAATGATATACACACATTTAAGATCGATTTGTTATA

TCCAGCATTCTGTTAATTAAATAGCCGACTATATTTTATTTATTCATTTACTCTACATAAACTTTTAGGGTTGGATTTG

TGCTTTTTCGAATAATTGTCGCAGAGAGTCATATCTAACAGAGTAGGTCACTCTCCTAATTTTAGGTTTAACCACTTTA

TTGCTCATCACTCCAAAGAGATATTTGAGACAAGTCCCCGGACGTTCACAGCCATGCTCTTCGAGAGTTTTGACCTTGT

CTCGGCGTTGGCGACGCTGGCTGCGTGTTTAGTGTCCATGGCACTTCTTCTGGCCGTGTCCCAGCAGCTCTGGCAGCTG

AGGTGGACCGCAACACGGGACAAGAGCTGCAAGCTGCCCATGCCCAAGGGCTCCATGGGGTTCCCCATCATCGGAGAAA

CATGCCACTGGTTCTTTCAGGTAAGCGCTCATTCTCCTTGTGCCTTTCCAAGTTTCCAGTCTCCCTGCTGTCGCACGCA

CAGCGCTCCGGCGCGCCAGACACCGCGGGCTCGGCGCATATAGTTTTTCCTGCAGGTAACTGGGTTGTTAAAGACACTT

CAAGAGTAGGATAACGTCCACTAATGCTTTTCGCACGTGTGCACTCGTGAGCAATATTGCGCTTTAAAATAAGAACAGC

TTGCAGGTTAGTTATTCCCACGTGTGTAATTTCTTCCGTGCCATATTTCCATGGTGTTAATAAGCTGTCTGTTTTAGAT

AGCCGTCAGTTTGTTTAATACAGCCAGTTCTCAATCCACGTGGAACAATCTGTTTGCGGCTGCCAAGGAAGGCGCACT

CCGTTCCAGAAATATTGTAGTCCTTGGCTTCCGATGCGCGCGAGAACACAGCCACTGAGCACCGCTTGTCTAAGCGCGT

CTGTTTGCGCGCGGGGTAAGATTGATCGCGAGCCGGGGGCAGGTATAGTGTCGCGCTCTTATTGAGCTTCAGTGTAATT

ATGCGCGCCGGTTTCCCAAAAAAGCCACCGTGTTGTGAATGATGCCGTGCCGTTCTGTCATGATACCCAGACAGCGCGT

GGGGCGCATAAAGTGCACGCTGGAGGTCTCTCTCTCTCTCTCTCACTGTCCGCAGTAAGAAAGTTTGTGAACGCTCAGA

TGAATAAAAAAAAGTTATAAATTAAGATCAGACCAGCGGCTATAATGTGGTATTTTGAAAAGAGTTTATTACGCGAACC

TTAGCTGTAAAATTGTCTCAAGAATTCTTTCCATATTAAATCCACGGTGCACATTGGGATGTCTCTGGACACTGGTTTG

CAGTAGCTGGAGTGGGTTTCTTTAAAGGGAGATTTGCGGAGATTCAGTAGGGAGGAATGTCCTGGTCTGAGTGGTGTAC

AGATCAAATTCTCTGGTCAGGAATAGATTCTCCAAACTTGCTGGAAATCCAGAAGAGAATTTCCATGCAGCTGGCAGGC
GCTCTGCAAAAACAAACAGACTTGACTGCATCTCAGAGTTATATGTGTAAAATCAATAGGATTTGGAGCGGTCCATTTG
AGCCAAACTTTTGTCATTTTTTATTTCCACAGCTCAGTTCAGGACTGCACGGCGACTGAAAGTTACAGTGCAGCCTGAA
ATGTTATCTCTTATTTGTGCTTCTGAAAACATTCAGTGCTAATTAACATCTGCATACGGAGGTTTAAGACGGGTCCTTT
AACTTTGCCATTTAGCTCATTTTGTGGTACTTGTTCTTCTGTCTTCTTAAAACTCAGCGGCAGATAAAACCATAAGTGG
GTCAAATCTATGATTGACAGATTTATGTATTCAGAGGAGTCTACATTTAAGTGCGTTCTCTTGCTGTGTGTCTGGCGTG
ATGAGTTGTGATATGATTCTGTATTTTGTCTGTGCATGGTTTTTGGGAGACCACATAATATCAGATCAGAGCTTTCTTT
TTTGTGCCACTCTCAACAGTCTTCCAGGTTTTGGCTGAGCTACCAATATGAGCT

Partial sequecne of mutated *cyp26b1* created by SGN

#7-1,#7-2 and #7-3

ATTCATAGAAACGCGCTTCACAGATCGAAATATTTGGCCATATCAATAATTTTGGCAAGGACTATCTGATATAATTAAG
AAAACACTGCCAGTTTATGAGGGATTTATTTACATTATTTTATAGAAATCGACTTATAACATCAACCCCCGAAAGTTGT
AAATATAAACGCAAAACAAATATGAAACGCGTAAGTCAATGAATGAATCGCTTACTCATGCAGTGTTCAGAGGACTGCT
GCTGCTGCTGCTGCTGCTGCTGCTGTGTGCAACAGATGCGCATCTGAACAATCAAAACATTTTGAAAAAGTCATGAACA
TATCGATTTAGCATAAGAGTCCGTAGCTTATATGATCCGGTAAAATGTAGAGTATTATTTCACATTTTTGTTGTGATGG
TTTTAAACCATCATTTGGGTAATCAGTGTACCTCAGTTTGAGCGCATTTTAAGTCCACAACATGAATTAACCCTGTGCG
TATTCCGCGAGTGGCAGGTGACAAGTTCCCGACCGTAAATGAGATTTGAAATATTGAAAGTGTTCCGCCCTCTCCCTCT
CTTTCTGTTTGGGCTC---2610bp---TCTTCCAGGTTTTGGCTGAGCTACCAATATGAGCT

Fig. S7 The SGN cleaves double-stranded DNA with nicked-structures into the ones with large deletion. a: the diagram of the double-stranded DNA fragment. The recognition sites of nicking endonulcease Nt.BstNBI were marked in red and the cutting positions were pointed by arrows. b: the cleavage products of SGN cutting were separated by 2% agarose gel. Lane 1: the nicked double-stranded DNA fragment without SGN; Lane 2: the nicked double-stranded DNA fragment with SGN; Lane M: DL-500 marker (500, 400, 300, 200, 150, 100 and 50 bp); Lane 3: the double-stranded DNA fragment without SGN; Lane 4: the double-stranded DNA fragment with SGN. *： denoting the the double-stranded DNA products with large deletion.

a

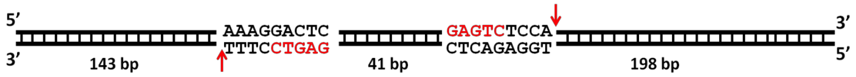

b

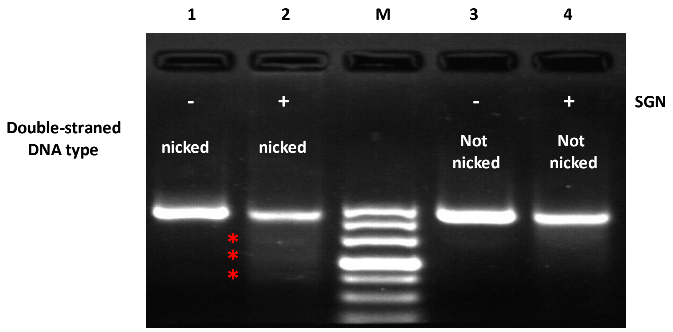

Supplement: Additional file 1: — Supplemental Table and Figures. Supplemental Table S1 and Figures S1–S7. (PDF 742 kb) [file 13059_2016_1038_MOESM1_ESM.pdf]
